# Mitochondrial DNA heteroplasmy in ovine fetuses and sheep cloned by somatic cell nuclear transfer

**DOI:** 10.1186/1471-213X-7-141

**Published:** 2007-12-21

**Authors:** Jörg P Burgstaller, Pamela Schinogl, Andras Dinnyes, Mathias Müller, Ralf Steinborn

**Affiliations:** 1 Institute of Animal Breeding and Genetics, University of Veterinary Medicine, Veterinaerplatz 1, 1210 Vienna, Austria; 2 Szent István University, Gödöllö, 2103, Hungary; 3 Agricultural Biotechnology Center, Genetic Reprogramming Group, Gödöllö, 2100, Hungary

## Abstract

**Background:**

The mitochondrial DNA (mtDNA) of the cloned sheep "Dolly" and nine other ovine clones produced by somatic cell nuclear transfer (SCNT) was reported to consist only of recipient oocyte mtDNA without any detectable mtDNA contribution from the nucleus donor cell. In cattle, mouse and pig several or most of the clones showed transmission of nuclear donor mtDNA resulting in mitochondrial heteroplasmy. To clarify the discrepant transmission pattern of donor mtDNA in sheep clones we analysed the mtDNA composition of seven fetuses and five lambs cloned from fetal fibroblasts.

**Results:**

The three fetal fibroblast donor cells used for SCNT harboured low mtDNA copy numbers per cell (A: 753 ± 54, B: 292 ± 33 and C: 561 ± 88). The ratio of donor to recipient oocyte mtDNAs was determined using a quantitative amplification refractory mutation system (ARMS) PCR (i.e. ARMS-qPCR). For quantification of SNP variants with frequencies below 0.1% we developed a restriction endonuclease-mediated selective quantitative PCR (REMS-qPCR). We report the first cases (n = 4 fetuses, n = 3 lambs) of recipient oocyte/nuclear donor mtDNA heteroplasmy in SCNT-derived ovine clones demonstrating that there is no species-effect hindering ovine nucleus-donor mtDNA from being transmitted to the somatic clonal offspring. Most of the heteroplasmic clones exhibited low-level heteroplasmy (0.1% to 0.9%, n = 6) indicating neutral transmission of parental mtDNAs. High-level heteroplasmy (6.8% to 46.5%) was observed in one case. This clone possessed a divergent recipient oocyte-derived mtDNA genotype with three rare amino acid changes compared to the donor including one substitution at an evolutionary conserved site.

**Conclusion:**

Our study using state-of-the-art techniques for mtDNA quantification, like ARMS-qPCR and the novel REMS-qPCR, documents for the first time the transmission of donor mtDNA into somatic sheep clones. MtDNA heteroplasmy was detected in seven of 12 clones tested, whereby all but one case revealed less than 1% mtDNA contribution from the nuclear donor cell suggesting neutral segregation.

## Background

Somatic cell nuclear transfer (SCNT) has been used to produce live clones in 14 mammalian genera ([1,2] and refs therein). Live animals were also cloned by interspecific (*Bos *[3]), and intersubspecific SCNT (mouflon [3], cattle [4], mice [5] and wild cat [6]). The transmission of donor-cell mtDNA to the clonal offspring was addressed in four mammalian species [4,5,7-11].

Mature oocytes in different mammalian species contain on average 1.74 to 9.5 × 10^5 ^mtDNA copies [12-14]. A threshold of about 100,000 mtDNA copy numbers must be exceeded for fertilisation to ensue in mouse, human and pig (reviewed in [15]). In contrast, mtDNA copy numbers in somatic cells vary substantially ranging from several hundred to several thousand [14,16-18]. Recipient oocytes have around 100 to 250 times more mtDNA copies than donor cells based on copy number ratios of 0.4% to 0.9% [4,5,8]. Therefore the resultant SCNT clones are assumed to have nuclear and mitochondrial genomes of different origins and to be slightly heteroplasmic based on the quantitative participation of mtDNA from the two partners and on equal replication of their mitochondrial genomes. Nucleus donor-derived mtDNA was transmitted to most cattle, mouse or pig clones [4,5,8,10,19,20], including the germline of one SCNT pig [10]. Quantitative studies on SCNT-derived fetuses and offspring revealed levels of heteroplasmy between 0 and 13% [4,5,8,19-21] indicating neutral transmission of mtDNAs of the nuclear donor and the oocyte recipient. However, higher contributions up to 40% attributed to a replicative advantage of donor mtDNA have also been observed [20]. These data demonstrate that donor mtDNA transmission is not prevented in SCNT and are concordant with the detection of somatic cell-derived mtDNA in offspring produced by assisted reproductive techniques like ooplasm transfer [22]. However, in Dolly and nine other cloned sheep donor mtDNA could not be detected [7].

The existence of heteroplasmic clones is contrary to the unimaternal inheritance of mtDNA during mammalian sexual reproduction [23]. The mechanism of paternal mitochondrial elimination involves the proteasome of oocytes, which recognizes mitochondria that have been ubiquitinated during spermatogenesis [24,25]. For sexually reproduced mammals there are few exceptions from this strict exclusion including reports of interspecies mouse hybrids [26], a human patient with mitochondrial myopathy [27,28], and the offspring of a single Small-tail Han sheep crossed to two rams of Dorset breed [29].

In this work, we re-investigated the issue of the failure to detect donor mtDNA transmission into cloned offspring in *Ovies aries *reported previously [7]. In addition to this previous report, our SNP quantification method included an intra-assay standard controlling for the detection limit reached in each run. To further increase discrimination sensitivity, we developed a restriction endonuclease-mediated selective quantitative PCR (REMS-qPCR) protocol. Both quantification approaches identified donor mtDNA transmission to ovine clones demonstrating the lack of a species-specific mechanism preventing transmission to SCNT sheep. In addition, we analysed complete mitochondrial genomes for amino acid changes being specific for high-level heteroplasmy found in another clone.

## Results

### Transmission of donor mtDNA to the clonal offspring

We investigated the transmission of nucleus donor cell mtDNA to twelve ovine SCNT clones (CA1 to CA7, CB1 to CB3 and CC1 and CC2; in total n = 7 fetuses and n = 5 lambs; Table 1), which were generated from three sources of primary fetal fibroblasts (A, B and C). Samples were taken from fetuses (cells or tissues), live lambs (blood and skin) and from a lamb put down *post partum *(several tissues) (Table 1).

**Table 1:** Heteroplasmy in tissues of ovine fetuses and sheep cloned by SCNT from fetal fibroblasts

**Clone**	**% donor mtDNA in tissues analysed**	**Source**
CA1	n.d. in blood, skin	lamb
CA2	blood (0.3), skin (0.3)	lamb
CA3	blood (0.6), skin (0.7)	lamb
CA4	n.d. in skin, tongue	fetus
CA5	brain (46.5), intestine (14.4), kidney (26.2), liver (11), lung (18.4), testis (6.8), tongue (10.4)	fetus
CA6	n.d. in amnion, blood, cerebellum, chorioallantois, heart, kidney, liver, lung, muscle, skin, small intestine, umbilicus	lamb
CA7	n.d. in placenta, skin, tongue	fetus
		
CB1	n.d. in cells at passage 2	fetus
CB2	cells at passage 2 (0.1)	fetus
CB3	cells at passage 2 (0.2)	fetus
		
CC1	cells at passage 0 (0.9)	fetus
CC2	skin (0.2), brain (0.3), hind limb muscle (0.2); n.d. in ileum, kidney, liver, lung, spleen, testis	lamb

n.d., not detectable

Culturing cells *in vitro *under standard, high-glucose conditions with sodium pyruvate and non-dialised fetal bovine serum supplementation was reported to reduce mtDNA copy number [30]. To estimate the expected levels of heteroplasmy in SCNT clones in case of neutral transmission of parental mtDNAs, we quantified the mtDNA copy numbers per cell for the three primary fetal fibroblast donors cultured for several passages under similar conditions before SCNT. Copy numbers obtained (donors A: 753 ± 54, B: 292 ± 33 and C: 561 ± 88) were comparable to those found in somatic cells of murine fetuses between days 8.5 and 13.5 *post conceptionem *(349 to 988; [14]) or in ovine fetal primary fibroblasts cultured long-term (genotype SFF1: 896 ± 55, [31]). However, they were lower than those found in earlier cultures derived from the latter genotype (SFF1: 4241 ± 411 to 1168 ± 76, [31]), in cultured goat fetal fibroblasts at different points in time (1129 ± 174 to 4276 ± 159, [31]) or in adult human primary fibroblasts (~1300 [32], 1600 to 2000, [33], 2400–6000 [16]).

**Figure 1:**
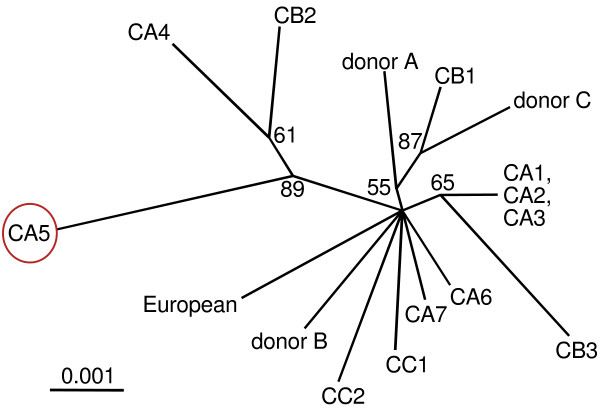
**The majority of the clonal offspring harbours different recipient oocyte-derived mitochondrial genotypes**. An unrooted phylogenetic tree derived by maximum likelihood using TREE-PUZZLE 5.2 with default settings from an alignment of concatenated mtDNA sequences (3806 nucleotides: control region, *MT-CO1*, *MT-CO3*, and *MT-ND4L*) of the three donor cell lines, their SCNT clones (CA1 to CA7, CB1 to CB3, and CC1 and CC2) and of an European mitochondrial reference genome (GenBank:AF010406). The scale bar represents 0.001 substitutions per site, and quartet puzzling values are shown (all are >50). The numbers at the nodes (quartet puzzling values) indicate the frequencies of occurrence for 1,000 replicate trees. Quartet puzzling support values provide an estimate of support of a given branch and can be interpreted in much the same way as bootstrap values. CA5 is the most divergent donor A-derived clone which is highlighted by the ellipse (see below for amino acid changes).

**Figure 2:**
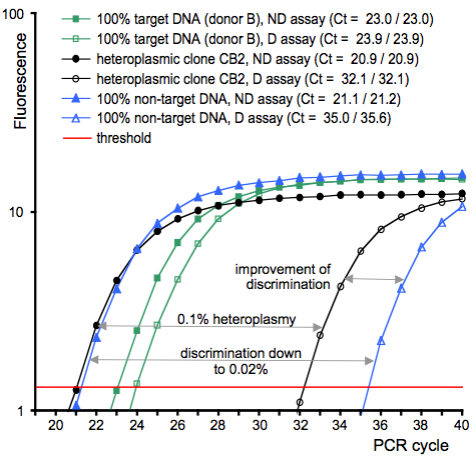
**The novel REMS-qPCR improves the quantitative detection limit for low-abundant point mutations**. This method requires pre-PCR cleavage of the high-abundant variant (non-target DNA here: recipient-oocyte mtDNA) and ARMS-qPCR (non-discriminative (ND) and discriminative (D) assays) for the low-abundant SNP variant (target here: donor mtDNA) at the mutated site. The amplification efficiency of the D assay was 91%. Without further optimization concerning conditions for enzymatic digestion, quantification by REMS-qPCR targeting a single donor-B-specific SNP reached a detection limit of 0.02%, i.e. a point mutation discrimination selectivity factor of 5 × 10^3^. It allowed detection of heteroplasmy (0.1%) in the donor B-derived clone CB2. This could not be detected by conventional ARMS-qPCR discriminating point mutations only down to 0.1% (see: illustrated improvement of discrimination). For clarity each plot is presented as the mean calculated from duplicate amplification reactions. Individual Ct values, i.e. PCR cycle numbers at which plots crossed an arbitrarily placed signal threshold, are given in the figure key. An independent technical replicate of this REMS-qPCR experiment demonstrated reproducibility of the method (data not shown).

To identify SNPs that could be used for detection of donor mtDNA transmission by ARMS-qPCR, we sequenced the mitochondrial control region and the mtDNA genes *MT-ND4L*, *MT-CO1 *and *MT-CO3 *for the somatic donors and their clones. The phylogenetic tree constructed with these sequences demonstrates a random maternal origin of the biological material (Figure 1). Only the recipient oocytes used to clone CA1, CA2 and CA3 were derived from the same maternal lineage or individual. This was additionally confirmed with sequences of the complete mitochondrial genome (see below). Donor mtDNA-specific polymorphisms were selected for assay design of ARMS-qPCR and the novel REMS-qPCR [Additional file 1]. We identified seven heteroplasmic sheep clones (n = 3 lambs, n = 4 fetuses) among the twelve screened. In three cloned lambs (CA2, CA3, CC2) we found at least two heteroplasmic tissues with 0.2 to 0.9% donor mtDNA (Table 1). In the heteroplasmic cloned fetuses we quantified low (0.1%, 0.2% and 0.9% in cells isolated from CB2, CB3 and CC1, respectively) or high (6.8% to 46.5% in tissues of CA5) levels of heteroplasmy (Table 1, Figure 2). There was no mtDNA substitution associated with transmission or non-transmission of donor mtDNA to the clones (Additional file 2, Table 2).

**Table 2:** Polymorphisms in the coding region of the mitochondrial genomes of donor A and its clones

**Sample**	**Gene**	**Base or amino acid change**	**Localisation of polymorphism**	**% in population (sequences tested)**	**Variation in human mtDNA**^5^
donor A	*MT-RNR2*	T1114C	domain 1 [69]	10 (n = 10)	n.a.
donor A	*MT-TF*	C41T	anticodon stem [70]	0.8 (n = 132)	n.a.
donor A	*MT-CYB*	Ile238Thr^3^	transmembrane domain 6^1^	18 (n = 28)	Ser/Pro/Phe
CA1-3^2^	*MT-ND6*	Val154Ile^3^	transmembrane domain 5^1^	≤ 13 (n = 8)	Val
CA5	*MT-CO1*	Met466Thr^3^	transmembrane domain 12^1^	< 5 (n = 21)	Met/Val
CA5	*MT-CO3*	Asn125Asp	longest extra-membranous loop^1,4^	< 5 (n = 21)	Asn
CA5	*MT-ND4L*	Met47Thr^3^	transmembrane domain 2^1^	< 5 (n = 21)	Met/Thr/Ile
CA5	*MT-RNR1*	A810T	domain 3 [71]	2.6 (n = 38)	n.a.
CA6	*MT-TG*	T134C	DHU loop [72]	9.1 (n = 11)	n.a.

n.a., not analysed

^1^According to Swiss Protein families database of alignments and hidden Markov models (Pfam)

^2^Recipient oocytes used to clone CA1, CA2 and CA3 are of the same mtDNA genotype

^3^Uncharged amino acids

^4^hydrophilic loop exposed on the intermembrane space and connecting helices III and IV [41]

^5^according to Human Mitochondrial Genome Database [40]

Chimerism in the blood of some bovine fetuses generated by *in vitro *techniques was attributed to maternal-fetal transfer of genetic material across an epitheliochorial placenta [34]. The two blood streams of all chorioallantoic placental types – epitheliochorial, synepitheliochorial, endotheliochorial and haemochorial – have areas of comparable proximity reducing the diffusion distance [35]. The reduction of interhaemal area varies in part with placental type [36]. In epitheliochorial (cow) and synepitheliochorial (sheep) placentae, capillary indenting of both trophoblast and uterine epithelium decreases the interhaemal distance [37]. Therefore, this issue was assessed in order to exclude the possibility of foster-mother mtDNA transfer. Four pairs of foster mother/cloned lamb were analysed (FM1/CA1, FM2/CA2, FM3/CA3 and FM4/CA6). Foster mother-specific ARMS-qPCR assays [Additional file 1] were designed on the basis of SNPs identified by sequencing the mitochondrial control region. We did not detect any foster-mother mtDNA in these four clones (see Table 1 for tissues analysed), at a detection limit ranging from 0.5 to 0.3%. Note that donor-cell-derived heteroplasmy in the clone CA2 (0.3%) was quantified at *15,633C*. For the heteroplasmic clones where foster-mother material was not available (clones CB3, CC1 and CC2), the assignment of mtDNA genotypes to somatic donor cells was based on quantification of heteroplasmy at two rare, donor-specific substitutions.

### High-level heteroplasmy in clone CA5

We addressed factors which could have been responsible for the high levels of heteroplasmy determined in CA5 (up to 46.5%, Table 1). First, we assessed the possibility of a foster mother-derived origin of this high level of heteroplasmy. Since biological material from the foster mother of clone CA5 was unavailable, we inferred donor transmission based on SNP analysis across the entire mitochondrial genome. All diagnostic SNPs (n = 34) for the donor and oocyte-derived mtDNAs were heteroplasmic in clone CA5, including some found at frequencies < 5% in the sheep population. Thus, an alternative origin of the heteroplasmy is exceedingly unlikely.

Next, we assessed the complete mtDNA genomes of donor A and its clones since polymorphisms in mtDNA can influence segregation of mammalian mtDNA independently from nuclear genes [38,39]. No polymorphism corresponded to either presence (clones CA2, CA3 and CA5) or absence (clones CA1, CA4, CA6 and CA7) of donor mtDNA (Table 2). The highly heteroplasmic clone CA5 possessed the most divergent mtDNA variant of the donor DA-derived clones. It differed in three rare amino acid changes not found in other sheep sequences (frequencies < 5%, Table 2). The change Asn125Asp in the gene *MT-CO3 *occurred at a site completely conserved in human (n = 2600, [40]) and highly conserved among 61 vertebrate species [Additional file 3]. With the exception of hedgehog (Asn125Thr) that seems to be most divergent in this extramembranous loop important for cytochrome *c *oxidase activity [41], the only variant occuring at this site in *Mammalia *is a Asn/Asp change seen in a few species ([Additional file 3] and data not shown). The other two amino acid changes found in the clone CA5 (Table 2) affected less conserved sites [Additional file 3].

## Discussion

Here we applied one (ARMS-qPCR) and developed another (REMS-qPCR) state-of-the-art techniques for mtDNA quantification to demonstrate donor mtDNA transmission in somatic sheep clones for the first time (in 7 out of 12 clones analysed). REMS-qPCR revealed a discrimination limit of 0.02%, and is thus one of the most sensitive methods for quantitative detection of SNP sequences. It offers increased power for discrimination of SNP sequences compared to ARMS-qPCR and their variations [4,8,11,42]. Application of REMS-qPCR was essential to identify low-level heteroplasmies of 0.1 and 0.2% in two of the three nuclear donor B-derived clones analysed in this work (clones CB2 and CB3). These two clones were derived from the donor B harbouring only about half of the mtDNA copies per cell determined for the nuclear donor A (A: 753 ± 54, B: 292 ± 33). The twofold higher mtDNA copy number found in the cells of donor A is concordant with slightly higher levels of heteroplasmy found in donor A-derived heteroplasmic clones, indicating neutral transmission of the donor mtDNA (clones CA2 and CA3: 0.3–0.7%). These levels of donor mtDNA-derived heteroplasmy are in agreement with the general picture that the majority of investigated SCNT-derived fetuses and offspring displays either undetectable levels of donor mtDNA or only mild heteroplasmy, which is consistent with neutral segregation of donor mtDNA [43]. It is highly likely that our improved SNP discrimination limits finally resulted in detecting donor mtDNA, which was not detected in an earlier study applying less discriminative methods [7].

The failure to detect donor mtDNA in seven other sheep cloned from a fetal fibroblast origin (PDFF2, [7]), i.e. the same source of somatic donor cells as used here, still remains unresolved. Again, a low mtDNA copy number per donor cell in combination with an insufficiently discriminative quantification method can not be excluded. Likewise, cryopreservation of the donor cell immediately before SCNT (PDFF2 in [44]) which did not allow the mitochondria to sufficiently recover from cryopreservation-induced oxydative stress or damage should be taken into account. Cryopreservation of oocytes, somatic and embryonic cells leads to a decrease of the mitochondrial membrane potential [45-47], which is an indicator of overall mitochondrial function. Moreover, cryopreservation is known to be responsible for the increased production of reactive oxygen species ([47,48] and refs. therein). Reactive oxygen species-induced oxidative stress leads to cell cycle arrest and changes in mtDNA copy number [49]. If donor cells were allowed to "recover" from cryopreservation over several passages before SCNT, the transmission of their mtDNA was not circumvented [8,50,51].

In this work all clones analysed were produced from early-passage fetal fibroblasts without cryopreservation.

Mammalian mtDNA segregation is controled by nuclear and mitochondrial genomes [52,53]. From previous work it can be concluded that different sources of recipient oocytes – genetically identical or random – can increase the chance to produce divergent segregation patterns of donor mtDNA in SCNT. Genetically identical recipient oocytes used to produce intersubspecific SCNT mice resulted in heteroplasmy levels of a few percent (including a single tissue with 13% heteroplasmy, [5]). In contrast, a study with genetically random-source recipient oocytes in cattle included two SCNT clones with a significant increase of donor mtDNA [20].

We also produced different patterns of donor mtDNA transmission using an identical nuclear background (donor A) in combination with recipient oocytes with different mtDNA. Beside undetectable (clones CA1, CA4, CA6 and CA7) or neutral transmission (clones CA2 and CA3 possessing the same mtDNA variant), a single case with a high degree of nuclear donor-derived heteroplasmy was observed (up to 46.5% in clone CA5, Figure 1 and Table 1). Heteroplasmies detected in some of the tissues analysed for the fetus CA5 are higher than those found in 18 heteroplasmic ovine embryos cloned by fusing enucleated oocytes of random genetic origin with ovine fetal primary fibroblasts (<8.7%, [11]). Our results show that high-level heteroplasmy can also occur in sheep following SCNT. Similar results have been obtained earlier for a bovine fetus and a calf cloned from cumulus cells (fetus: 39% (range: 25–51%), calf: 40% (range 8–59%), [20]). Authors attributed this high heteroplasmy to a replicative advantage of donor over recipient-oocyte mtDNA [20]. In addition to the latter report, this work is the first to provide mtDNA coding region sequences for a highly heteroplasmic SCNT clone and relates the obviously favoured proliferation of donor mtDNA to a divergent mitochondrial genotype (Figure 1, Table 2). Irrespective of the polymorphism frequency in the local sheep herds used as recipient-oocyte source or of evolutionary conservation which are not decisive criteria to classify a variant as mildly deleterious [54], we speculate that one or several predicted amino acid changes of this mtDNA variant could have caused suboptimal nucleo-mitochondrial interaction with the nucleus of donor A by affecting functions of the mitochondrially encoded protein subunits inside and/or outside of the mitochondrion [55].

A certain level of variability in mtDNA segregation has to be taken into account for embryo reconstruction [56]. However, we believe that the 'non-pathological' mtDNA variant of CA5 (its recipient-oocyte mtDNA) which encodes one or several amino acid changes represents a promising new genetic candidate to study mtDNA proliferation, nucleo-mitochondrial interaction or the selective preference of some cell types for a given mitochondrial genotype [39].

In this work mitochondrial genomes with up to about 0.2% sequence divergence were mixed by SCNT. Heteroplasmic mice carrying mitochondrial genotypes of *Mus musculus domesticus *and *Mus musculus brevirostris *(NZB mtDNA [57]) with a sequence divergence of 0.7% (GenBank:L07095 and J01420; 15 amino acid changes [30]) have been described as normal and healthy, as no overtly abnormal phenotype was observed [58]. The level of heteroplasmy in these mouse models did not appear to affect the capacity for oxidative phosphorylation at the level of the organelle [30]. However, differences were detected when physiological parameters of heteroplasmic mice carrying a mixture of the same mtDNA genotypes at levels between 19 and 56% were studied in greater detail [59].

## Conclusion

Following a report describing the potential of sheep SCNT embryos to harbour donor mtDNA throughout preimplantation development [11], this is the first report demonstrating donor mtDNA transmission to fetuses or sheep cloned by SCNT (7 out of 12 clones analysed). Our data are concordant with reported heteroplasmy in bovine, murine and porcine SCNT clones [5,8,10]. Thus, we demonstrate that there is no species-specific effect in sheep, which prevents transmission of nucleus donor-derived mtDNA into clonal offspring.

## Methods

### Biological material

SCNT was performed with MII stage oocytes derived from superovulated ewes and the following three donor cell lines at early passages: (i) fetal fibroblast cells at passage 2 of a day-35 Finn Dorset fetus (No. 7G65F4, denoted donor A; [60]), (ii) fetal fibroblast cells at passage 4–6 derived from a day-26 Black Welsh Mountain fetus (BLWF1, denoted donor B; [7,61]), and (iii) fetal fibroblast cells at passage 3 derived from a day-35 Black Welsh Mountain fetus (BW6F2, denoted donor C; [60]). SCNT was performed as reported [61] using serum deprivation to synchronise donor cells at the G0 phase of the cell cycle. Fibroblasts from day-35 Finn Dorset and day-26 Black Welsh Mountain fetuses were recovered as described [61] using standard, high-glucose cell culture conditions and medium supplementation with sodium pyruvate and non-dialised fetal calf serum.

In addition to the SCNT clones CA1-CA3, CA5 and CA6 derived from the primary cell line 7G65F4 (see above), we also analysed genetically engineered clones (CA4 and CA7) derived from cell lines with random integration or homologous targeting of a α(1,3)galactosyl transferase (*GGTA1*) gene construct, respectively [60]. The cloned lamb CC2 carries a prion protein (PrP) targeted gene deletion [60].

Early cloned fetuses (35 d: clones CB1, CB2, CB3, CC1; 49 d: clone CA5) were recovered by intentional termination of pregnancy. The other two cloned fetuses analysed died in utero (clone CA7, 130 d) or were stillborn (clone CA4, 148 d).

In addition, we analysed DNA of four recipient foster cows (FM1 to FM4) which carried the clones CA1, CA2, CA3 and CA6, respectively, to exclude a contribution of the recipient ewe's mtDNA to the clone (mimicking heteroplasmy) due to placental leakage.

Breed information of the biological material is given in Additional file 4.

### DNA isolation

Cell, blood and tissue samples were shipped on dry ice and stored at -80°C. DNA isolation was performed using the NucleoSpin^® ^Tissue kit (Macherey & Nagel, Dueren, Germany).

### Short tandem repeat (STR) analysis

We confirmed the nuclear genetic identity of all clones using a panel of 11 standardized STR loci recommended by the National Institute for Agricultural Research (INRA, Paris, France) for parentage control in the sheep (data not shown). Fluorescence data collection and analysis was performed as described [4].

### Sequence analysis

A primer walking approach was used for cycle sequencing of PCR products performed in a 96-well format with the BigDye™ terminator chemistry (Applied Biosystems, Foster City, USA) as described [62].

### Demonstration of mtDNA genotype diversity using phylogenetic analysis

The phylogeny of mtDNA control region sequences (excluding the tandem repeat element IV not present in the Asian mtDNA) was reconstructed with the program TREE-PUZZLE, version 5.2 [63] – a program package for quartet-based maximum-likelihood phylogenetic analysis. The phylogenetic tree constructed with default settings was graphically displayed using TREEVIEW, version 1.6.6 [64].

### Quantification of mtDNA copy number in donor cells by quantitative qPCR

For mtDNA quantification the donor cells were used at similar passage numbers as used for SCNT (2, 2 and 3 for donors A, B and C, respectively). The mtDNA copy number per cell was estimated by TaqMan qPCR taking as reference the copy number of the diploid nuclear genome. The *MT-CO1 *gene and the single copy gene coding for the ovine gonadotropin-releasing hormone receptor (*GnRHR*; [65]) were used as mitochondrial and nuclear target sequences, respectively, in TaqMan qPCR (RTPrimerDB assay IDs: 3804 and 3805; [66]). In detail, 334-bp *MT-CO1 *and 290-bp *GnRHR *fragments were designed on the basis of the GenBank sequences AF010406 and L42937, respectively. Amplicons generated from genomic DNA (primers: TTGGAGCCCCTGATATAGCATT and GAGAGAAGGAGAAGTACGGCAGTAA (*MT-CO1*), and AAAACACTTGACTTTAGCCAACCTG and TGGTTTACCTGTGGTCCAGCA (*GnRHR*)) were purified with the NucleoSpin^® ^Extract II kit (Macherey-Nagel, Düren, Germany). The copy number of each amplicon was calculated from the mean of three OD_260 _values measured for three different amplicon dilutions on the U-3000 spectrophotometer (Hitatchi, Tokyo, Japan). Amplicons were titrated and tenfold serially diluted in 5 ng/mL sheared salmon sperm DNA (Invitrogen, Lofer, Austria). These concentration standards were used in TaqMan qPCR to determine the mtDNA copy numbers per cell in each sample. Quantitative qPCR was performed on the ABI Prism 7900 HT Sequence Detection System (Applied Biosystems, Foster City, USA). The copy number of the nuclear and mitochondrial target sequences for the sample DNAs was concluded by the ABI Prism SDS software 2.3 (Applied Biosystems, Foster City, USA) from the respective standard curve which was generated from a series of tenfold standard dilutions co-amplified in each run. The linear regression analysis of all standard curves showed a high correlation (correlation coefficient ≥ 0.997). Quantification was performed in two technical replicates, which started with the generation of the amplicon concentration standard and ended with duplicate amplifications of the mitochondrial and nuclear targets of each sample performed in independent runs. Finally, the mtDNA copy number per diploid nuclear genome was expressed as mean ± standard deviation calculated from the technical replicates.

### Amplification refractory mutation system quantitative PCR (ARMS-qPCR)

The ratio of mixed SNP alleles, i.e. the level of heteroplasmy, was determined by ARMS-qPCR [4,8,67,68]. This method is based on qPCR quantification of the total mtDNA content and of the minor mtDNA variant of each sample using a non-discriminative and a discriminative assay, respectively. The percentage of donor mtDNA was calculated according to the standard curve method [4]. The sensitivity of the ARMS-qPCR assay, i.e. the limit for detection of a portion of the matching allele above the background caused by the delayed illegitimate amplification from the mismatched allele, was controlled by the use of a homogenous template carrying the alternative base for the SNP under study. Homoplasmic mismatching ovine DNA or if not available for a specific SNP locus, a synthetic amplicon (MWG Biotech, Ebersberg, Germany) mixed with 5 ng/mL sheared salmon sperm DNA were used to control for the amplification from the mismatching allele. Oligonucleotide sequences of the assays are provided in [Additional file 1].

### Restriction endonuclease-mediated selective quantitative PCR (REMS-qPCR)

REMS-qPCR is based on pre-PCR cleavage of the non-target sequence (here: recipient-oocyte mtDNA) at a given biallelic SNP site (here: position 16,284) and subsequent selective amplification of the low-abundant target mtDNA (here: mitochondrial genotype of the donor). In detail, 40 ng DNA was digested for 5 minutes with FastDigest™ Mph 1103I (MBI Fermentas, St. Leon-Rot, Germany) and purified with the MSB Spin PCRapace Kit (Invitek, Berlin, Germany). The low-abundant point mutation was subsequently quantified at position 16,284 by ARMS-qPCR (assays ND4 and D12, [Additional file 1]) to reduce unspecific amplification from the incompletely digested non-target mtDNA variant.

### Nucleotide sequences

Nucleotide sequences were deposited with the GenBank. Their accession numbers are listed in Additional file 4.

## List of abbreviations used

SCNT: Somatic cell nuclear transfer; ARMS-qPCR: Amplification refractory mutation system quantitative real-time PCR; REMS-qPCR: Restriction endonuclease-mediated selective quantitative real-time PCR.

## Authors' contributions

JPB, PS and RS conceived and designed the experiments. JPB and RS developed the REMS-qPCR. JPB and PS performed the experiments. AD contributed tissues. MM and RS supervised the interpretation of data. RS supervised the experimental work and wrote the paper. All authors analysed the data, commented on and approved the manuscript.

## Supplementary Material

Additional file 1 Non-discriminative (ND) and discriminative (D) primer/TaqMan probe sets for detection of mtDNA heteroplasmy by ARMS-qPCR and REMS-qPCR. Click here for file

Additional file 2 Genetic variation in putative elements of the control region detected in mtDNA genotypes involved in this study. Click here for file

Additional file 3 **Extent of conservation of the amino acid changes found in mitochondrial genes of clone CA5**. Partial amino acid sequence alignment of the mtDNA genes *MT-CO1*, *MT-CO3 *and *MT-ND4L *from 61 vertebrate species. Click here for file

Additional file 4 GenBank accession numbers for partial or complete mtDNA sequences of somatic donor cells, their clonal offspring and foster mothers. Click here for file

## References

[B1] Berg DK, Li C, Asher G, Wells DN, Oback B (2007). Red deer cloned from antler stem cells and their differentiated progeny. Biol Reprod.

[B2] Shi D, Lu F, Wei Y, Cui K, Yang S, Wei J, Liu Q (2007). Buffalos (Bubalus bubalis) cloned by nuclear transfer of somatic cells. Biol Reprod.

[B3] Campbell KH, Alberio R, Choi I, Fisher P, Kelly RD, Lee JH, Maalouf W (2005). Cloning: eight years after Dolly. Reprod Domest Anim.

[B4] Steinborn R, Schinogl P, Wells DN, Bergthaler A, Mueller M, Brem G (2002). Coexistence of Bos taurus and B. indicus Mitochondrial DNAs in Nuclear Transfer-Derived Somatic Cattle Clones. Genetics.

[B5] Inoue K, Ogonuki N, Yamamoto Y, Takano K, Miki H, Mochida K, Ogura A (2004). Tissue-specific distribution of donor mitochondrial DNA in cloned mice produced by somatic cell nuclear transfer. Genesis.

[B6] Gomez MC, Pope CE, Giraldo A, Lyons LA, Harris RF, King AL, Cole A, Godke RA, Dresser BL (2004). Birth of African Wildcat cloned kittens born from domestic cats. Cloning Stem Cells.

[B7] Evans MJ, Gurer C, Loike JD, Wilmut I, Schnieke AE, Schon EA (1999). Mitochondrial DNA genotypes in nuclear transfer-derived cloned sheep. Nat Genet.

[B8] Steinborn R, Schinogl P, Zakhartchenko V, Achmann R, Schernthaner W, Stojkovic M, Wolf E, Müller M, Brem G (2000). Mitochondrial DNA heteroplasmy in cloned cattle produced by fetal and adult cell cloning. Nat Genet.

[B9] St John JC, Moffatt O, D'Souza N (2005). Aberrant heteroplasmic transmission of mtDNA in cloned pigs arising from double nuclear transfer. Mol Reprod Dev.

[B10] Takeda K, Tasai M, Iwamoto M, Akita T, Tagami T, Nirasawa K, Hanada H, Onishi A (2006). Transmission of mitochondrial DNA in pigs and progeny derived from nuclear transfer of Meishan pig fibroblast cells. Mol Reprod Dev.

[B11] Lloyd RE, Lee JH, Alberio R, Bowles EJ, Ramalho-Santos J, Campbell KH, St John JC (2006). Aberrant nucleo-cytoplasmic cross-talk results in donor cell mtDNA persistence in cloned embryos. Genetics.

[B12] Smith LC, Thundathil J, Filion F (2005). Role of the mitochondrial genome in preimplantation development and assisted reproductive technologies. Reprod Fertil Dev.

[B13] May-Panloup P, Vignon X, Chretien MF, Heyman Y, Tamassia M, Malthiery Y, Reynier P (2005). Increase of mitochondrial DNA content and transcripts in early bovine embryogenesis associated with upregulation of mtTFA and NRF1 transcription factors. Reprod Biol Endocrinol.

[B14] Cao L, Shitara H, Horii T, Nagao Y, Imai H, Abe K, Hara T, Hayashi J, Yonekawa H (2007). The mitochondrial bottleneck occurs without reduction of mtDNA content in female mouse germ cells. Nat Genet.

[B15] Bowles EJ, Campbell KH, St John JC (2007). Nuclear transfer: preservation of a nuclear genome at the expense of its associated mtDNA genome(s). Curr Top Dev Biol.

[B16] Shmookler Reis RJ, Goldstein S (1983). Mitochondrial DNA in mortal and immortal human cells. Genome number, integrity, and methylation. J Biol Chem.

[B17] King MP, Attardi G (1989). Human cells lacking mtDNA: repopulation with exogenous mitochondria by complementation.. Science.

[B18] Frahm T, Mohamed SA, Bruse P, Gemund C, Oehmichen M, Meissner C (2005). Lack of age-related increase of mitochondrial DNA amount in brain, skeletal muscle and human heart. Mech Ageing Dev.

[B19] Hiendleder S, Zakhartchenko V, Wenigerkind H, Reichenbach HD, Bruggerhoff K, Prelle K, Brem G, Stojkovic M, Wolf E (2003). Heteroplasmy in bovine fetuses produced by intra- and inter-subspecific somatic cell nuclear transfer: neutral segregation of nuclear donor mitochondrial DNA in various tissues and evidence for recipient cow mitochondria in fetal blood. Biol Reprod.

[B20] Takeda K, Akagi S, Kaneyama K, Kojima T, Takahashi S, Imai H, Yamanaka M, Onishi A, Hanada H (2003). Proliferation of donor mitochondrial DNA in nuclear transfer calves (Bos taurus) derived from cumulus cells. Mol Reprod Dev.

[B21] Theoret CL, Dore M, Mulon PY, Desrochers A, Viramontes F, Filion F, Smith LC (2006). Short- and long-term skin graft survival in cattle clones with different mitochondrial haplotypes. Theriogenology.

[B22] Barritt JA, Brenner CA, Malter HE, Cohen J (2001). Mitochondria in human offspring derived from ooplasmic transplantation. Hum Reprod.

[B23] Breton S, Beaupre HD, Stewart DT, Hoeh WR, Blier PU (2007). The unusual system of doubly uniparental inheritance of mtDNA: isn't one enough?. Trends Genet.

[B24] Sutovsky P, Moreno RD, Ramalho-Santos J, Dominko T, Simerly C, Schatten G (1999). Ubiquitin tag for sperm mitochondria. Nature.

[B25] Sutovsky P, Moreno RD, Ramalho-Santos J, Dominko T, Simerly C, Schatten G (2000). Ubiquitinated sperm mitochondria, selective proteolysis, and the regulation of mitochondrial inheritance in mammalian embryos. Biol Reprod.

[B26] Gyllensten U, Wharton D, Josefsson A, Wilson AC (1991). Paternal inheritance of mitochondrial DNA in mice. Nature.

[B27] Schwartz M, Vissing J (2002). Paternal inheritance of mitochondrial DNA. N Engl J Med.

[B28] Kraytsberg Y, Schwartz M, Brown TA, Ebralidse K, Kunz WS, Clayton DA, Vissing J, Khrapko K (2004). Recombination of human mitochondrial DNA. Science.

[B29] Zhao X, Li N, Guo W, Hu X, Liu Z, Gong G, Wang A, Feng J, Wu C (2004). Further evidence for paternal inheritance of mitochondrial DNA in the sheep (Ovis aries). Heredity.

[B30] Battersby BJ, Shoubridge EA (2001). Selection of a mtDNA sequence variant in hepatocytes of heteroplasmic mice is not due to differences in respiratory chain function or efficiency of replication. Hum Mol Genet.

[B31] Bowles EJ, Lee JH, Alberio R, Lloyd RE, Stekel D, Campbell KH, St John JC (2007). Contrasting effects of in vitro fertilization and nuclear transfer on the expression of mtDNA replication factors. Genetics.

[B32] Timmermans EC, Tebas P, Ruiter JP, Wanders RJ, de Ronde A, de Baar MP (2006). Real-time nucleic acid sequence-based amplification assay to quantify changes in mitochondrial DNA concentrations in cell cultures and blood cells from HIV-infected patients receiving antiviral therapy. Clin Chem.

[B33] Legros F, Malka F, Frachon P, Lombes A, Rojo M (2004). Organization and dynamics of human mitochondrial DNA. J Cell Sci.

[B34] Hiendleder S, Bebbere D, Zakhartchenko V, Reichenbach HD, Wenigerkind H, Ledda S, Wolf E (2004). Maternal-fetal transplacental leakage of mitochondrial DNA in bovine nuclear transfer pregnancies: potential implications for offspring and recipients. Cloning Stem Cells.

[B35] Wooding FBP, Flint APF, GE L (1994). Placentation. Marshall’s Physiology of Reproduction, Part I.

[B36] Enders AC, Carter AM (2004). What can comparative studies of placental structure tell us?--A review. Placenta.

[B37] Leiser R, Pfarrer C, Abd-Elnaeim M, Dantzer V (1998). Feto-maternal anchorage in epitheliochorial and endotheliochorial placental types studied by histology and microvascular corrosion casts. Trophoblast Res.

[B38] Jenuth JP, Peterson AC, Shoubridge EA (1997). Tissue-specific selection for different mtDNA genotypes in heteroplasmic mice. Nat Genet.

[B39] Moreno-Loshuertos R, Acin-Perez R, Fernandez-Silva P, Movilla N, Perez-Martos A, de Cordoba SR, Gallardo ME, Enriquez JA (2006). Differences in reactive oxygen species production explain the phenotypes associated with common mouse mitochondrial DNA variants. Nat Genet.

[B40] Ingman M, Gyllensten U (2006). mtDB: Human Mitochondrial Genome Database, a resource for population genetics and medical sciences. Nucleic Acids Res.

[B41] Lincoln AJ, Donat N, Palmer G, Prochaska LJ (2003). Site-specific antibodies against hydrophilic domains of subunit III of bovine heart cytochrome c oxidase affect enzyme function. Arch Biochem Biophys.

[B42] Urata M, Wada Y, Kim SH, Chumpia W, Kayamori Y, Hamasaki N, Kang D (2004). High-sensitivity detection of the A3243G mutation of mitochondrial DNA by a combination of allele-specific PCR and peptide nucleic acid-directed PCR clamping. Clin Chem.

[B43] Hiendleder S, Zakhartchenko V, Wolf E (2005). Mitochondria and the success of somatic cell nuclear transfer cloning: from nuclear-mitochondrial interactions to mitochondrial complementation and mitochondrial DNA recombination. Reprod Fertil Dev.

[B44] Schnieke AE, Kind AJ, Ritchie WA, Mycock K, Scott AR, Ritchie M, Wilmut I, Colman A, Campbell KHS (1997). Human factor IX transgenic sheep produced by transfer of nuclei from transfected fetal fibroblasts. Science.

[B45] Petrenko AY (1992). A mechanism of latent cryoinjury and reparation of mitochondria. Cryobiology.

[B46] Ahn HJ, Sohn IP, Kwon HC, Jo do H, Park YD, Min CK (2002). Characteristics of the cell membrane fluidity, actin fibers, and mitochondrial dysfunctions of frozen-thawed two-cell mouse embryos. Mol Reprod Dev.

[B47] Jones A, Van Blerkom J, Davis P, Toledo AA (2004). Cryopreservation of metaphase II human oocytes effects mitochondrial membrane potential: implications for developmental competence. Hum Reprod.

[B48] Kopeika J, Zhang T, Rawson DM, Elgar G (2005). Effect of cryopreservation on mitochondrial DNA of zebrafish (Danio rerio) blastomere cells. Mutat Res.

[B49] Lee CF, Liu CY, Hsieh RH, Wei YH (2005). Oxidative stress-induced depolymerization of microtubules and alteration of mitochondrial mass in human cells. Ann N Y Acad Sci.

[B50] Zakhartchenko V, Durcova-Hills G, Stojkovic M, Schernthaner W, Prelle K, Steinborn R, Mueller M, Brem G, Wolf E (1999). Effects of serum starvation and re-cloning on the efficiency of nuclear transfer using bovine fetal fibroblasts. J Reprod Fertil.

[B51] Zakhartchenko V, Alberio R, Stojkovic M, Prelle K, Schernthaner W, Stojkovic P, Wenigerkind H, Wanke R, Duchler M, Steinborn R, Mueller M, Brem G, Wolf E (1999). Adult cloning in cattle: Potential of nuclei from a permanent cell line and from primary cultures. Mol Reprod Dev.

[B52] Battersby BJ, Loredo-Osti JC, Shoubridge EA (2003). Nuclear genetic control of mitochondrial DNA segregation. Nat Genet.

[B53] Takeda K, Takahashi S, Onishi A, Hanada H, Imai H (2000). Replicative advantage and tissue-specific segregation of RR mitochondrial DNA between C57BL/6 and RR heteroplasmic mice. Genetics.

[B54] Bandelt HJ, Salas A, Bravi CM (2006). What is a 'novel' mtDNA mutation--and does 'novelty' really matter?. J Hum Genet.

[B55] Sadacharan SK, Singh B, Bowes T, Gupta RS (2005). Localization of mitochondrial DNA encoded cytochrome c oxidase subunits I and II in rat pancreatic zymogen granules and pituitary growth hormone granules. Histochem Cell Biol.

[B56] Meirelles FV, Smith LC (1998). Mitochondrial genotype segregation during preimplantation development in mouse heteroplasmic embryos. Genetics.

[B57] Yonekawa H, Moriwaki K, Gotoh O, Miyashita N, Migita S, Bonhomme F, Hjorth JP, Petras ML, Tagashira Y (1982). Origins of laboratory mice deduced from restriction patterns of mitochondrial DNA. Differentiation.

[B58] Sligh JE, Levy SE, Waymire KG, Allard P, Dillehay DL, Nusinowitz S, Heckenlively JR, MacGregor GR, Wallace DC (2000). Maternal germ-line transmission of mutant mtDNAs from embryonic stem cell-derived chimeric mice. Proc Natl Acad Sci U S A.

[B59] Acton BM, Lai I, Shang X, Jurisicova A, Casper RF (2007). Neutral mitochondrial heteroplasmy alters physiological function in mice. Biol Reprod.

[B60] Denning C, Burl S, Ainslie A, Bracken J, Dinnyes A, Fletcher J, King T, Ritchie M, Ritchie WA, Rollo M, de Sousa P, Travers A, Wilmut I, Clark AJ (2001). Deletion of the alpha(1,3)galactosyl transferase (GGTA1) gene and the prion protein (PrP) gene in sheep. Nat Biotechnol.

[B61] Wilmut I, Schnieke AE, McWhir J, Kind AJ, Campbell KHS (1997). Viable offspring derived from fetal and adult mammalian cells. Nature.

[B62] Burger PA, Steinborn R, Walzer C, Petit T, Mueller M, Schwarzenberger F (2004). Analysis of the mitochondrial genome of cheetahs (Acinonyx jubatus) with neurodegenerative disease. Gene.

[B63] Schmidt HA, Strimmer K, Vingron M, von Haeseler A (2002). TREE-PUZZLE: maximum likelihood phylogenetic analysis using quartets and parallel computing. Bioinformatics.

[B64] Page RDM (1996). TREEVIEW: An application to display phylogenetic trees on personal computers. Computer Applications in the Biosciences.

[B65] Campion CE, Turzillo AM, Clay CM (1996). The gene encoding the ovine gonadotropin-releasing hormone (GnRH) receptor: cloning and initial characterization. Gene.

[B66] Pattyn F, Robbrecht P, De Paepe A, Speleman F, Vandesompele J (2006). RTPrimerDB: the real-time PCR primer and probe database, major update 2006. Nucleic Acids Res.

[B67] Thomassin H, Kress C, Grange T (2004). MethylQuant: a sensitive method for quantifying methylation of specific cytosines within the genome. Nucleic Acids Res.

[B68] Steinborn R, Zakhartchenko V, Wolf E, Müller M, Brem G (1998). Non-balanced mix of mitochondrial DNA in cloned cattle produced by cytoplast-blastomere fusion. FEBS Lett.

[B69] Mears JA, Sharma MR, Gutell RR, McCook AS, Richardson PE, Caulfield TR, Agrawal RK, Harvey SC (2006). A structural model for the large subunit of the mammalian mitochondrial ribosome. J Mol Biol.

[B70] Wakita K, Watanabe Y, Yokogawa T, Kumazawa Y, Nakamura S, Ueda T, Watanabe K, Nishikawa K (1994). Higher-order structure of bovine mitochondrial tRNA(Phe) lacking the 'conserved' GG and T psi CG sequences as inferred by enzymatic and chemical probing. Nucleic Acids Res.

[B71] Hickson RE, Simon C, Cooper A, Spicer GS, Sullivan J, Penny D (1996). Conserved sequence motifs, alignment, and secondary structure for the third domain of animal 12S rRNA. Mol Biol Evol.

[B72] Lauber J, Marsac C, Kadenbach B, Seibel P (1991). Mutations in mitochondrial tRNA genes: a frequent cause of neuromuscular diseases. Nucleic Acids Res.

