# Supplementary material for: Mitochondrial DNA heteroplasmy in ovine fetuses and sheep cloned by somatic cell nuclear transfer
Source: BMC Dev Biol. 2007 Dec 21;7:141. doi: 10.1186/1471-213X-7-141 (PMC2323970; doi:10.1186/1471-213X-7-141)
Supplement: Additional file 3 — Extent of conservation of the amino acid changes found in mitochondrial genes of clone CA5. Partial amino acid sequence alignment of the mtDNA genes MT-CO1, MT-CO3 and MT-ND4L from 61 vertebrate species. [file 1471-213X-7-141-S3.DOC]

***MT-CO1*** ***MT-CO3*** ***MT-ND4L***

*Ovis aries* MGSFISLTAVMLMIFIIWEAF LAPTPELGGCWPPTGIHPLNPLE MLSLFILATLMILNSHFTLAS

*Ovis aries – clone CA5* ..........T.......... ...................D... ..........T..........

*Artibeus jamaicensis* .............V.M..... .........Y...A..T.....D ......M..IT...T.L....

*Balaenoptera musculus* ..................... ....................... .....VMMA.T.........N

*Balaenoptera physalus* ..................... ................R...... .....V..A.T..S......N

*Bos taurus* .............V....... ....................... .....VM.A.T..........

*Canis familiaris* ...............M..... ................I...... .....VMMSVT...N.L....

*Cavia porcellus* ...........V.V.M..... .........F...A..N....M. ...T.VFGS.VM....YL.SF

*Cebus albifrons* V......A..I....M..... .....QT..H......F....M. ......MM.T.T..M.LM.MY

*Ceratotherium simum* .............V.M..... .....................M. ......M..M.V.........

*Chalinolobus tuberculatus* ..........V..V.MV.... .........Y...A..T....M. V....V.T...V.TINL..TN

*Dasypus novemcinctus* ...............M..... ................N...... .......S.....SM...M.M

*Didelphis virginiana* I.........I..V....... ....................... ......FMAA..THF.MFSI.

*Dugong dugon* ..........I..V.MV.... ...................D.M. .......G..LC..M....SA

*Echinops telfairi* V.........I....MT.... ................T...... ....Y..NSTLA.SM....Y.

*Echinosorex gymnura* ...........ILV.MV.... .........V...A..N...... .......MSIST..FNNS..M

*Equus asinus* ...............M..... ....................... .....VM..MVV..T......

*Equus caballus* ...............M..... ....................... .....VM..M.V..T......

*Erinaceus europaeus* .............V.L...R. .V..T....Y...QELNH.TH.K ..ATY.FIS.VS..M...MTY

*Felis catus* .............V.MV.... ................I...... ......MMAVA...N.L....

*Gorilla gorilla* V..............M..... .....Q..AH......T...... ......M....T..T..L..N

*Halichoerus grypus*  .............V.M..... ................T....M. .....VMM.VT...N......

*Hippopotamus amphibius* ..........V..V....... ................N...... ......M...I...A......

*Homo sapiens* V..............M..... .....Q...H......T...... ......M....T..T.SL..N

*Hylobates lar* V..............M..... .....Q...H......T...... ......MS...A..T.SL.IN

*Isoodon macrourus* I.........I.......... .............V......... .......M..L.SHF.MYSM.

*Lama pacos* V.........I..V..V.... ....................... .....VM.S....ST......

*Loxodonta africana* .......V..I..V.M..... ........SY...V.VY...... .....T.NA.LS..MN...ST

*Macaca sylvanus* ..........I...YM..... .....C...H......I...... AM....MTA.IAS.T.SP.IN

*Macropus robustus* I.........I..V....... ....................... .......MA.L.SHF.MFS..

*Mus musculus* ..........LI...M..... .V..HD..........S...... V.....MTSVTS...NSMSSM

*Myoxus glis* ...........I.M.MV.... ................TLF.... ...M..INSAI...ML.S.SF

*Nycticebus coucang* I.........V....MV.... ................N.....D ...M...VS.TTM.L.....N

*Ochotona collaris* ...........I...M..... .............V..T...... .....M...IVS..MN..ISF

*Ornithorhynchus anatinus* L...V......V...M..... ................S...... ...I..MVA.IL.HH.LNSTM

*Orycteropus afer* ..........II......... ................F....M. .......G.T.A..M....MT

*Oryctolagus cuniculus* ...........V...M..... ................N...... .....V.I..TA..T....SF

*Pan paniscus* V..............M..... .....Q...H......T...... ......MT...T..T.SL..N

*Pan troglodytes* V..............M..... .....Q...H......T...... ......M....T..T.SL..N

*Papio hamadryas* .........TI...YM..... .....R..CH......T...... .M....MTAV.AS.A.SP.IN

*Phoca vitulina* .............V.M..... ................T....M. .....VMM.VT...N......

*Physeter catodon* ..................... ................C..D.FK A.....FT..TT..L.....N

*Pongo pygmaeus* A..............M..... .....Q...H......I...... ......MI...T..T.SL..N

*Pongo pygmaeus abelii* A..............M..... .....Q...H......T...... ......MI...T..T.SL..N

*Pteropus dasymallus* ..........I..V.M..... ....................... .....VTMS.T....NLV...

*Pteropus scapulatus* ..........I..V.M..... ....................... .....VTMA.T....NLV...

*Rattus norvegicus* ..........LV...M..... .V..HD..........T...... .....VMTSTST...NSMISM

*Rhinoceros unicornis* .............V.MV.... .....................M. ......M..M.V........I

*Sciurus vulgaris* ...........I...M..... .............V..N...... ...M..MT..AT..T..MVSF

*Soriculus fumidus* ..........VI.V.MV.... ....H....Y...A..N...... ..T...MSSI.V..M......

*Sus scrofa* ..................... ....................... ......MS..I...T.....N

*Tachyglossus aculeatus* L..........V...MV.... ................S...... ......MVS.IL.HH.LNSTM

*Talpa europaea* .............V.M..... ....H....F...A......... ..A....S.I....I......

*Tarsius bancanus* .............V.M..... ................Y...... ...M..MTS.T...L....SN

*Thryonomys swinderianus* ...........M...M..... .........H......N...... ...M...S..TM..L.YVSFM

*Trichosurus vulpecula* I.........I..V.T..... ....H.................. .....VMMA.L.SHF.MFST.

*Tupaia belangeri* ...............M..... ................T...... .....V.SSILV..N.LI.TA

*Ursus americanus* ...............M..... ........A.......T...... .....VML.VTV..N.....N

*Ursus arctos* ...............M..... ........A.......T...... .....VML.VTV..N.....N

*Ursus maritimus* ...............M..... ........A.......T...... .....VML.VTV..N.....N

*Volemys kikuchii* ..........LI.V.M..... .V..HD......T...S...... .....M...ITP..T.SMIMF

*Vombatus ursinus* I.........I..V..V.... ....................... .......MA.L.SHF.MLSV.

Additional file 3

Extent of conservation of the amino acid changes found in mitochondrial genes of clone CA5. Partial sequence alignment of the mtDNA genes *MT-CO1*, *MT-CO3* and *MT-ND4L* (amino acids 457-477, 107-129 and 38-58, respectively) from 61 vertebrate species including 57 mammals and 4 marsupials. Boxes designate changes in the recipient cytoplast-derived haplotype of the sheep clone CA5. The conserved hydrophilic peptide on the intermembrane space of cytochrome c oxidase is underlined in the partial alignment of *MT-CO3*. The extent of conservation was assessed with the mtSNP Database mtSAP evaluation approach (Tanak*a et a*l*. Ann N Y Acad Sc*i 2004**, 10**11:7-20).
